# Telocytes damage in endometriosis-affected rat oviduct and potential impact on fertility

**DOI:** 10.1111/jcmm.12427

**Published:** 2014-11-11

**Authors:** Xiao-Jun Yang, Jian Yang, Zhen Liu, Gang Yang, Zong-Ji Shen

**Affiliations:** a Department of Obstetrics and Gynecology, The First Affiliated Hospital of Soochow UniversitySuzhou, China; b Lab Center, Medical College of Soochow UniversitySuzhou, China

**Keywords:** telocytes, endometriosis, infertility, fertility disorder, tubal ectopic pregnancy, interstitial cells, rat model, oviduct, fibrosis

## Abstract

Women with endometriosis (EMs) have unexplained infertility. The recently identified telocytes (TCs) might participate in the maintenance of structural and functional integrity of oviduct tissue, but so far the involvement of TCs in EMs-affected oviduct tissue and potential impact on fertility capacity remain unknown. By an integrated technique of haematoxylin and eosin staining, *in situ* immunohistochemistry and double-labelled immunofluorescence staining and electron microscopy approach, TCs were studied in the autotransplantation Sprague–Dawley rat model of EMs-affected oviduct tissue and in sham control, respectively, together with determination of iNOS, COX-2, LPO and estradiol. TCs were found in perivascular connective tissue and smooth muscle bundles in sham oviduct, with typical ultrastructural features (a slender piriform/spindle/triangular cell body, and one or more extremely long prolongations, emerged from cell bodies and extend to various directions), and specific immunophenotype of CD34-positive/vimentin-positive/c-kit-negative. However, in EMs-affected oviduct tissue (grade III), extensive ultrastructural damage (degeneration, discontinue, dissolution and destruction), significant decrease or loss of TCs and interstitial fibrosis were observed, together with elevated level of iNOS, COX-2, LPO and estradiol, thus suggestive of inflammation and ischaemia-induced TCs damage. Based on TCs distribution and intercellular connections, we proposed that such damage might be involved in structural and functional abnormalities of oviduct, such as attenuated intercellular signalling and oviduct contractility, impaired immunoregulation and stem cell-mediated tissue repair, 3-D interstitial architectural derangement and tissue fibrosis. Therefore, TCs damage might provide a new explanation and potential target for EMs-induced tubal damage and fertility disorders.

## Introduction

Endometriosis (EMs) is an inflammatory, ischaemic, hyperestrogenic condition associated with many clinical manifestations, particularly in women of childbearing age 1,2, such as chronic pelvic pain, dysmenorrhea, and sub- or infertility 3. The critical pathological changes were the disturbance of pelvic micro-environments, which produce an excessive amount of inflammatory factors, oxidative stressor and estradiol 2,4. Many classical theories tried to explain reason of EMs-associated decreased fertility or infertility. However, women with minimal and mild asymptomatic EMs still show complications with unexplained infertility and this remains a challenging issue, even in the absence of macroscopic pelvic alterations. On the other hand, the observed chronic salpingitis in EMs cases 5, and dysperistalsis of utero-tubal smooth muscle in early stage of EMs 6, strongly suggested oviduct roles in contribution to EMs-associated sub- or infertility.

Telocytes (TCs; previously considered as interstitial cajal-like cells, ICLC) exist in interstitial space of many normal tissues of mammalian and human organs 7–27, including endometrium 23, myometrium 24 and fallopian tube 25–27. Based on morphology and location in normal tissue, TCs have been proposed to possess potential multiple biological functions. And in a very few reports, TCs damage was observed in cardiac, skin and Crohn's disease 28–31. Previously, we reported that ICLC was decreased in women with EMs and tubal ectopic pregnancy 32. In addition, decrease of this kind of interstitial cells was associated with attenuated rabbit oviduct motility 33. Nevertheless, the exact ultrastructural alterations of TCs population in EMs-affected oviduct tissue remain unclear.

We suggested that, chronic exposure to pelvic micro-environments, which is characterized by overproduced inflammatory factors 2,4 (inducible nitric oxide synthase, iNOS; Cyclooxygenase-2, COX-2), oxidative stressor (lipid peroxide, LPO) and estradiol, might induce oviduct TCs damage and is then involved in structural and functional oviduct abnormalities, such as the observed dysmotility of oviduct 6, until finally contributing a significant role in EMs-associated tubal factor sub- or infertility. To test this hypothesis, we aimed to extend most of current literatures, which only focus on TCs in normal tissue, and conduct a comparative study of oviduct TCs, together with determination of iNOS, COX-2, LPO and estradiol, respectively, in EMs-affected oviduct tissue and in sham group. Such knowledge will be helpful to elucidate structural alterations of oviduct tissue underlying TCs damage and potential functional consequence on reproduction, with the aim of providing a potential target for genetic and pharmaceutical interventions.

## Materials and methods

### Animals

Three-month-old virgin female Sprague–Dawley rats (200–250 g) were used, all with regular 4- to 5-day estrous cycle before and after surgery, as determined by vaginal cytology 34. All rats were maintained at least 10 days under specific pathogen-free conditions with food and drinking water provided *ad libitum* before experiments. Rats were obtained from the Medical Experimental Animal Administrative Committee of Soochow university (animal certification number: 0102261), and handling procedures meet the guidelines of the Institutional Ethics Review Board of Soochow university.

### Animal model

The autotransplantation rat model of EMs-affected oviduct tissue was surgically constructed in mature female rats in oestrus 35,36. Briefly, the rats were anaesthetized with pentobarbital (50 mg/kg i.p.; Fuyang Pharmaceutical Factory, Fuyang city, China) prior to laparotomy with a low midline incision. A 1-cm segment of the right side of uterine horn was dissected and cut into two pieces longitudinally (≈4 × 4 mm). Then, two pieces of tissue masses were transplanted through interrupted sutures (4-0 Vicryl Rapide, Ethicon Endo-Surgery Inc., Cincinnati, Ohio, USA), with endometrial side towards both surface of contralateral mesosalpinx, respectively, and adjacent to the arteries that irrigate the oviduct. Rats in the sham group received control surgery with removal of the uterine horn and blank sutures, without any tissue masses. Then, followed with incision closure and bred in the same conditions until 2 months, as endometriotic vesicles appear to reach their maximal size at about 7.5 weeks post-surgery 35,36.

### Tissue harvesting and histology assessment

At 2 months, all rats were killed and oviduct segment with grade III ectopic endometriotic vesicles was harvested 37: the implant formed a cyst with fluid, and its major diameter of the vesicle was larger than 4 mm (similar to, or larger than, the initial size of the implant). Freshly dissected oviduct segments (1 cm^3^) were fixed in 4% formalin and embedded in paraffin. Transverse serial sections (5 μm) were processed for haematoxylin and eosin, *in situ* immunohistochemistry (IHC) and immuofluorescent staining. The rest of fresh tissue (1 mm^3^) was processed for transmission electron microscopy (TEM). Healthy oviduct taken from the sham group served as control.

### *In situ* IHC for TCs

Sections from sham group (5 μm) were subjected to procedures for *in situ* IHC staining. The primary antibodies were rabbit anti-rat polyclonal CD34 (1:100; cat. no. BA0532), mouse anti-rat monoclonal vimentin (1:100; cat. no. BM0135), rabbit anti-rat polyclonal c-kit (1:100; cat. no. BA0467-1; all provided by Boster, Wuhan, China). Images of the same areas of interest in consecutive sections were observed by light microscope. Omission of the primary antibodies served as the negative controls.

### *In situ* fluorescent IHC

Firstly, for quantitative determination of cytotoxic substance, single-labelled fluorescent IHC staining was used. Briefly, sequential sections (5 μm) from both groups were exposed to primary antibodies: rabbit anti-rat polyclonal iNOS (1:100; cat. no. sc-649), mouse anti-rat polyclonal COX-2 (1:100; cat. no. sc-166475), rabbit anti-rat polyclonal LPO (1:100; cat. no. sc-134849), and rabbit anti-rat polyclonal estradiol (1:100; cat. no. BA3399; all provided by Santa Cruz Biotechnology, Santa Cruz, CA, USA). Then FITC-goat anti-rabbit/mouse IgG (1:100; cat. no. BA1105/BA1101; all provided by Boster) was added. Finally, sections were coverslipped with antifade mounting medium (1:1000; cat. no. P0126; Beyotime, Shanghai city, China). Immunofluorescence intensity was quantitatively analysed by laser confocal scanning microscopy (TCS-SP2; Leica Lasertechnik, Heidelberg, Germany). Total fluorescent intensity per selected area was calculated by multiplying the number of pixels/area with the area mean intensity. More than 500 cells obtained from three separate microscopic fields were analysed for each marker 38.

Secondly, for further distinguishing, localization and precise counting of TCs, *in situ* double-labelled immuofluorescent staining was applied. Briefly, sequential 5-μm sections from both groups were exposed to the primary antibodies in pairs (CD34 *versus* vimentin, c-kit *versus* vimentin): CD34 (1:100), vimentin (1:100), c-kit (1:100). Then CY3-goat anti-rabbit IgG for CD34 (1:50; cat. no. BA1032), FITC-goat antimouse IgG for vimentin (1:50; cat. no. BA1101), TRITC-goat anti-rabbit IgG for c-kit (1:50; cat. no. BA1090) were added (all provided by Boster). Finally, counterstained with DAPI (1:50; cat. no. C1002) and mounted with antifade medium (1:1000; cat. no. P0126; both provided by Beyotime). Sections were then observed with fluorescence microscope (Olympus BX51, Tokyo, Japan). The number of TCs was identified in ten EMs-affected and ten sham samples, respectively, in a double-blinded manner (one section per sample), by correlating unique morphology (piriform/spindle/triangular-shaped cell body and extremely long and thin prolongations) with immunofluorescence, together with well-defined nuclei under 10 randomly selected microscopic high-power fields in the merged images per section (40*10 original magnification), and statistically analysed 29,30.

### Ultrastructure observation

Fresh oviduct fragments (1 mm^3^) from both groups were subjected to routine epon-embedding procedure 39. Ultra-thin sections (∽60 nm) of the selected areas were then rendered for TEM (Hitachi H-600, Hitachi Co., Tokyo, Japan) at 60 kV and photographed.

### Statistical analysis

All values were expressed as mean ± SD. *T*-test was performed with SPSS (version 13; SPSS Inc., Chicago, IL, USA). *P* < 0.05 was considered statistically significant.

## Results

### Routine pathological observation

**Fig 1:**
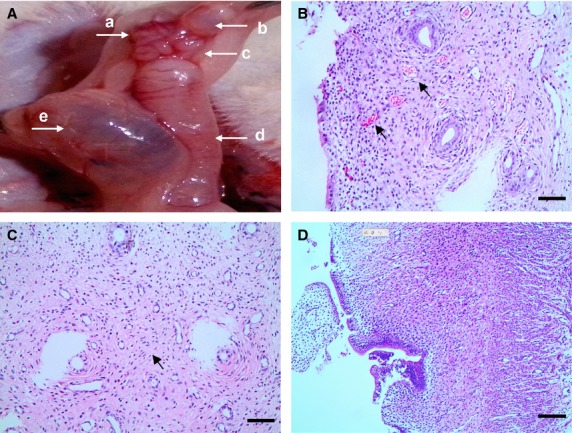
Surgically induced rat model of EMs-affected oviduct by tissue autotransplantation in mesosalpinx, scale bar = 100 μm. (A) Macroscopic ectopic endometrial vesicle invades serous membrane of mesosalpinx and oviduct tissue in the experimental group, with diameter larger than 4 mm (black arrow), classified as grade III according to Quereda *et al*. (a) ovarian tissue; (b) ectopic endometrial vesicle; (c) oviduct tissue; (d) the left side of uterine horn; (e) local swelling and effusion after removal of the right side of uterine horn. (B) Hyperplasia and disturbance of capillaries (black arrows), indicated non-specific tissue reaction against the invasion of exogenous endometrial glands into oviduct wall. (C) Increased infiltration of lymphocytes and increased contents of fibre (black arrow), with abnormal hyperplasia of small vessels in EMs-affected oviduct wall, indicate chronic inflammation and interstitial fibrosis. (D) Normal oviduct tissue from the sham control.

Macroscopically, ectopic endometriotic vesicles of various sizes invaded the serous membrane of mesosalpinx and/or surface of oviduct segment in experimental group, and appeared as oval shaped, slightly yellow, transparent, fluid filled, with capillary vessels on their thin surface (Fig.1A). Ten rats display cyst 4.22–7.57 mm in diameter (grade III) 37 and were selected for further study; meanwhile, none of the rats in sham group developed any cyst. Microscopically, EMs-affected oviduct tissue was characterized by (*i*) hyperplasia and disturbance of capillaries within the wall (Fig.1B); (*ii*) increased lymphocyte infiltration and increased contents of fibre, suggestive for chronic inflammation and interstitial fibrosis (Fig.1C). However, tissue structures unchanged in sham group (Fig.1D).

### *In situ* IHC

**Fig 2:**
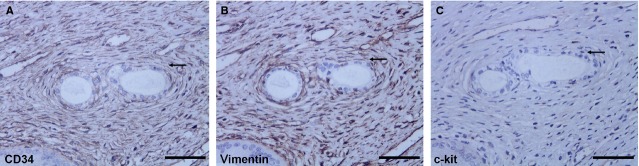
*In situ*IHC of CD34, vimentin and c-kit on serial slides from the sham control (black arrow); scale bar = 200 μm. (A) One TCs-like cell with CD34 (+), displayed a slender cell body and one or more cellular prolongations, located around capillaries. (B and C) Vimentin (+) and C-kit (−) cell in the same area of serial slides.

In particular areas on consecutive sections from sham oviduct, these cells with similar TCs morphology: stellate-shaped cells with prolonged cell body located around the vessels, demonstrated positive expression for CD34 (Fig.2A) and vimentin (Fig.2B), while negative for c-kit (Fig.2C). The specific morphology and expression patterns of these cells show that they are presumably TCs.

### *In situ* fluorescent IHC

**Fig 3:**
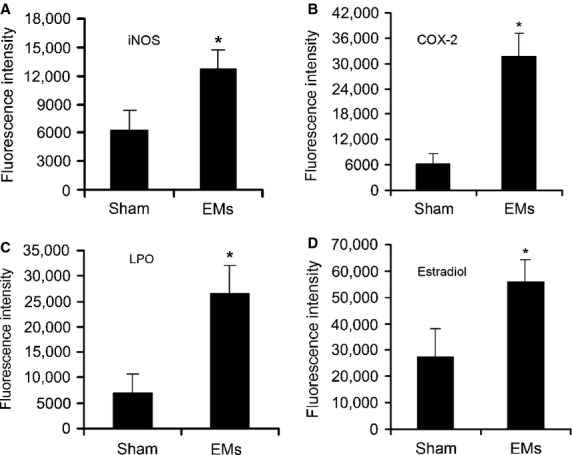
Toxic substance in EMs-affected oviduct tissue significantly higher than that of sham control. (A) iNOS, (B) COX-2, (C) LPO, (D) estradiol. *P < 0.05 *versus* sham control. Error bars = SD. (For the sake of clarity, only half of the error bars are shown).

Firstly, in EMs-affected oviduct tissue, significant up-regulation of all the markers was found as compared with the sham group, respectively: iNOS (*P* = 0.000; Fig.3A), COX-2 (*P* = 0.000; Fig.3B), LPO (*P* = 0.000; Fig.3C) and estradiol (*P* = 0.002; Fig.3D).

**Fig 4:**
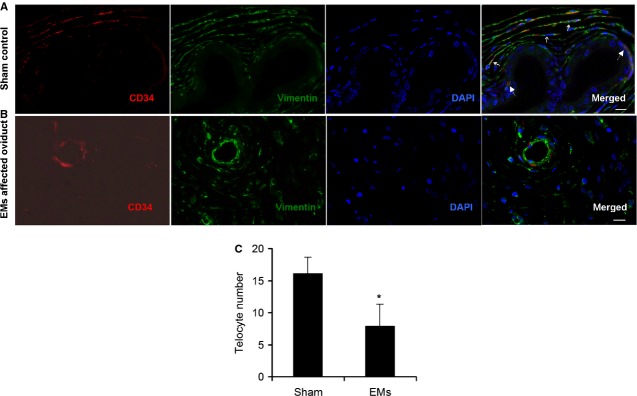
Representative double-labelled immunofluorescence images. Nuclei are counterstained with DAPI (blue). Images of c-kit negative were not shown here; scale bar = 20 μm. (A) CD34 (red) in moniliform appearance cells overlying vimentin (green) cells in sham control (three small solid arrows), confirmed the presence of telocytes (TCs) and telopodes (Tps) around capillaries, immunophenotype is CD34 (+)/vimentin (+)/c-kit (−). Two large dotted arrows indicated CD34 (+) capillary cells. (B and C) The number of CD34 (+)/vimentin (+) cells with typical TCs morphology and well-defined DAPI nuclei, decreased significantly, scarce or even undetectable (*P* = 0.000) in EMs-affected oviduct. *P < 0.05 *versus* sham control. Error bars = SD. (For the sake of clarity, only half of the error bars are shown).

Secondly, in sham group (Fig.4A), with the aid of fluorescence, we confirmed the existence of TCs with typical characteristic appearance, located around capillaries with one or more extremely long/thin cellular prolongations. Positive co-expressed CD34 (red) and vimentin (green) were found both in cell bodies and prolongations, overlapped each other with vimentin reactivity mainly located within prolongations and seemingly higher at the ends of prolongations. Meanwhile, negative staining of c-kit (images not shown), consistent with *in situ* IHC (Fig.2) and further confirmed immunophenotype of oviduct TCs in rat. However, in sections from EMs-affected tissue, quantitative analysis demonstrated that the number of CD34 (+)/vimentin (+) cells with typical TCs morphology and well-defined DAPI nuclei decreased significantly, TCs were sparse or even completely undetectable (*P* = 0.000; Fig.4B and C).

### Ultrastructure observation

**Fig 5:**
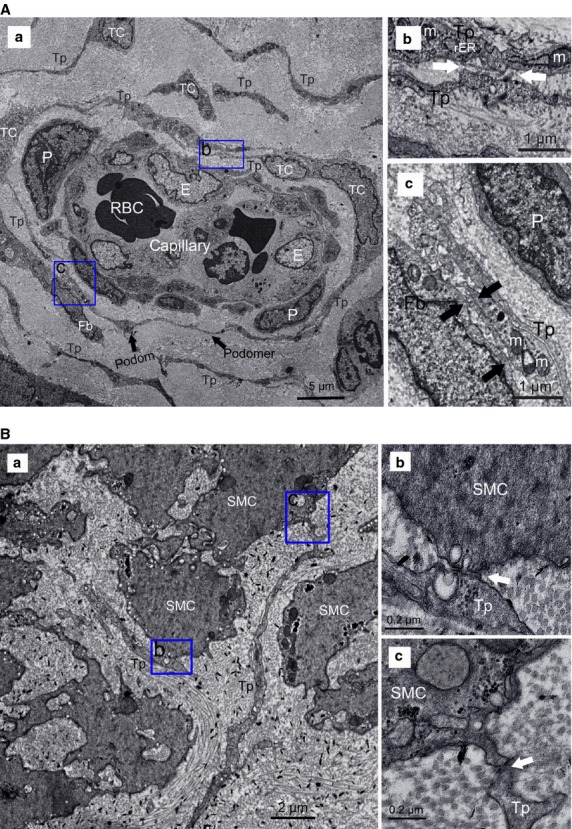
Normal telocytes (TCs) with their telopodes (Tps), surrounding capillaries or scattered between smooth muscle bundles. (A) TCs around capillaries. (a) two or three layers of TCs formed a sheath around vascular endothelial cells (E) with their Tps, which composed of podom and podomer (black arrows), with pericytes (P) between them, Tps formed an almost complete circle to enwrap the capillaries. The organelles, such as mitochondria (M), rough endoplasmic reticulum (rER), cytoskeletal elements, can be observed. (b and c) Higher magnifications of the boxed areas; (b) TCs frequently established homocellular junctions with their Tps (white arrows); (c) heterocellular contacts between TCs and fibrocyte (Fb; black arrows). (B) TCs among smooth muscle cells (SMC). (a) Tp display close contact with SMC. (b and c) Higher magnifications of the boxed areas, show microvesicles visible in synaptic cleft (white arrows).

Telocytes with specific ultrastructural features were identified in sham oviduct, appeared as a slender piriform/spindle/triangular-shaped cell body, with one or more extremely long, thin, very sinuous cellular prolongations (telopodes, Tps), emerged from cell body and extend to various directions. More detailedly, Tps are made by an alteration of thin segments (podomers) and thick segments (podoms). In addition, Tps accommodate the organelles, such as mitochondria, rough endoplasmic reticulum, cytoskeletal elements, caveolae and microvesicles (Fig.5A and B).

**Fig 6:**
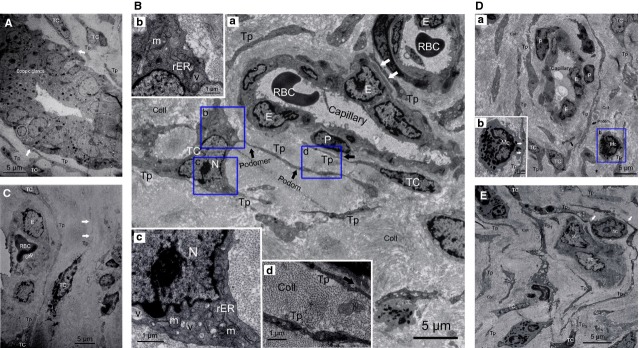
Telocytes (TCs) and telopodes (Tps) damage in EMs-affected oviduct tissue. (A) Ectopic endometriotic glands with abundant secretory granules, with close contact with Tps (white arrows). (B) Severely damaged TCs around capillaries, accompanied by nearly normal endothelial cells (E). (a) Loss of TCs network integrity and swollen cell junctions (white arrows). (b–d) Higher magnifications of the boxed areas; (b and c) damage of organelles, swollen cell nucleus (N) and mitochondria (m), rough endoplasmic reticulum (rER) dilatation, cytoplasmic vacuolization (v). (d) Cytoplasmic vacuolization within Tps, together with excessive amount of collagen fibres (Coll). All suggested EMs-induced TCs degeneration, tissue fibrosis and functional insufficiency. (C) Degeneration, discontinue, dissolution, destruction of TCs and Tps (white arrows), or even completely absent around capillary, together with severe damage of endothelial (E) and other type of cells. (D) TCs damage. (a) Slightly damaged TCs, with excessive amount of collagen fibril (Coll), (b) higher magnification of the boxed area, Tps made synapse to mast cell (MC; white arrows), indicated chronic inflammation and tissue fibrosis. (E) The damaged TCs and a group of putative normal stem cells (SC) make a possible SC niche, together with excessive amount of collagen fibres (Coll). The cluster of SC is surrounded by Tps, with intercellular contact between them (white arrows).

Telocytes frequently established homocellular contacts with their Tps, or make close contact with various types of vicinity cells through heterocellular junctions. TCs were resident dominantly around capillaries; two or three layers of TCs run parallel to each other and/or formed a sheath with their long prolongations enfolding the vascular endothelial cells, together with homocellular junctions through their Tps, or heterocellular junctions with fibrocyte and pericytes (Fig.5A). Moreover, TCs also scattered between smooth muscle bundles, often came in contact with smooth muscle cells (SMC), with microvesicles contained in Tps and synaptic cleft (Fig.5B). Meanwhile, TCs made heterocellular synapse to mast cells (MCs) with their Tps and potentially participated in immunoreactions (Fig.6D). TCs also surrounded stem cell (SC) niches with Tps and heterocellular contacts (Fig.6E).

Conversely, in EMs-affected oviduct tissue, which contained typical ectopic endometriotic glands with abundant secretory granules (Fig.6A), TCs display cell degeneration, discontinue, dissolution, destruction or even completely absent (Fig.6B–E), include loss of organelles, swollen cell nucleus and mitochondria, cytoplasmic vacuolization, endoplasmic reticulum dilatation, and swollen cell junctions, suggestive of cell functional insufficiency. This further proved the marked decreases or loss of TCs in EMS-affected oviduct (Fig.4B and C). In addition, abundant collagen fibres (Fig.6B, D and E) indicated development of tissue fibrosis. Interestingly, ultrastructural damage of TCs was sometimes accompanied by normal endothelial cells and SC (Fig.6B and E). And furthermore, TCs with nearly normal ultrastructure can also be observed around microvessels (Fig.6D), despite a generally decreased trend of cell count.

## Discussion

Endometriosis is a hyperestrogenic gynaecological disease characterized by the presence of endometrium outside the uterine cavity, affects an estimated 8–10% of women in their reproductive age and causes fertility problems in industrialized countries 1,3. Generally, typical pathophysiology was chronic inflammatory, ischaemic and hyperestrogenic state within pelvic micro-environments 2–4. Current knowledge on EMs-related fertility disorders remains insufficient, although multifactorial pathogenesis has been proposed. In women with advanced macroscopic pelvic lesions, mechanical alterations might adversely affect tubal peristalsis and subsequent oocyte pickup and transport. However, increasing evidence indicated that women with minimal and mild asymptomatic EMs also have unexplained sub- or infertility, even in the absence of any macroscopic anatomical pelvic abnormalities 3. Interestingly, the findings of chronic salpingitis 5, and dysperistalsis of utero-tubal smooth muscle in early stage of EMs 6, strongly suggested potential role of oviduct in EMs-related fertility problems, which arise from a subset of clinically affected oviduct tissue. Nevertheless, the autotransplanted rat model demonstrated ectopic endometriotic vesicles macroscopically (Fig.1A), chronic inflammation and interstitial fibrosis microscopically (Figs1B–C and 6A, B, D and E), together with overproduced iNOS, COX-2, LPO and estradiol in oviduct tissue (Fig.3A–D). Thus, resembled clinic physiopathology of EMs and provided an ideal animal model for infertility investigations.

Conventionally, stromal cells in oviduct tissue mainly include myocyte, dendritic cells, macrophages, MCs, plasma cells, eosinophils, lymphocytes, plus interstitial cells of Cajal (ICC), which were the pacemaker cells regulating oviduct rhythmic peristalsis 40, and fibroblasts, which were considered the principal effector cells involved in tissue fibrosis 30. Various types of cells play their respective roles and participate in reproductive process. Here, we examined the newly discovered TCs in disease-affected and -unaffected oviduct specimens. In normal oviduct, TCs distributed proximal to capillaries, among smooth muscle bundles or adjacent to SC (Figs5A, B and 6B–E), and show immunophenotype of CD34 (+)/vimentin (+)/c-kit (−) (Figs2 and 4A), thus confirming previous descriptions both ultrastructurally and phenotypically 7–27. However, immunophenotype of TCs varies with the organ and/or the animal species examined 26, possibly because of the existence of subpopulations of TCs. For example, in uterine tissue, TCs with c-kit (−)/vimentin (+) 41, c-kit (+)/vimentin (+), c-kit (+)/CD34 (+) were found 42, and in human dermal tissue, TCs were c-kit (−)/CD34 (+)/CD31 (−) 29,30. Others indicated that ICC was c-kit (+)/CD34 (−), while TCs were c-kit (−)/CD34 (+) 10,43. Such differences might be the basis of regional-specific TCs roles 44. As TCs do not possess a unique antigenic profile, currently, for TCs immunodiagnostics, the firmly reported positive CD34 from different organs, in combination with c-kit, vimentin and PDGFRa, remains the best available choice 31,44.

Nevertheless, most of current studies were focused on TCs in normal tissues, except a very few reports in cardiac, skin and Crohn's disease 28–31. Previously, we have shown by immunochemistry that oviduct ICLC was decreased in women with EMs and tubal ectopic pregnancy 32. We supposed that in EMs, regardless of whether or not accompanied by macroscopic pelvic lesions, the toxic micro-environments 2,4 might cause TCs damage and then might be involved in structural and functional abnormalities of oviduct. Such a hypothesis was strongly supported by the present results. Herein, we extend our previous findings and clearly describe presumably for the first time that in EMs-affected oviduct tissue, TCs display secondary ultrastructural damage (Fig. 6A–E) and significant cell loss (Fig.4B and C), complicated with development of tissue fibrosis (Fig.6). And accompanied by overproduced iNOS, COX-2, LPO and estradiol in oviduct tissue (Fig.3A–D), which might play important role in TCs damage. Furthermore, there is seemingly a positive trend of ultrastructural abnormalities and TCs reduction with the severity of pelvic EMs (data not shown). Interestingly, the assumption of reactive oxygen species-induced TCs damage has also been supposed in human skin systemic sclerosis 29. Nevertheless, as accompanied by normal endothelial and SC (Fig.6B and E), it is difficult to explain why TCs were important target cells and seemingly less tolerant to those critical damaging factors in the disturbed local pelvic milieu.

Recent genetic and proteomic analysis showed that lung TCs were completely different from fibroblasts 45,46, and the role of mechanical sensing, mechanochemical conversion, tissue homeostasis and remodelling/renewal, intercellular signalling and SC niche modulation has been suggested for lung TCs 46. In uterine tissue, TCs can influence the contractile activity of SMC, and TCs differed with pregnant states in telopodal width and podomic thickness, which was considered to be related to their function 47. Previously, similar TCs damage was described in experimental myocardial infarction, where cardiac TCs were significantly decreased 28, in dermal cellular network of skin systemic sclerosis, where they display ultrastructural damage, progressive reduction and loss of TCs 29,30, and loss of TCs in fibrotic lesions of Crohn's disease 31. However, there is still lack of sufficient and direct evidence about TCs involvement in oviduct diseases. We proposed that TCs damage might contribute to structural abnormalities of oviduct, involved in oviduct dysfunction in women with EMs, probably *via* the bio-functions that have been proposed based on their distribution and ultrastructure in different organs, as follows, although none of them has been certainly proved yet.

Firstly, homocellular contacts between TCs themselves (Fig.5A) and heterocellular junctions with various oviduct interstitium components, such as MCs (immunocytes; Fig.6D), SC (Fig.6E), SMC (Fig.5B), suggested that oviduct TCs might be involved in intercellular information exchange between various stromal cells, or represent a ‘functional unit’ by participating in making a primitive nervous system through TCs–exosomes–gap junctions–cytoskeleton 48. In particular, close contact between TCs and MCs in present study (Fig.6D) and previous literatures 25,26 suggests that TCs were involved in MCs-mediated immunoregulation/immunosurveillance. Actually, TCs display specific direct (homocellular or heterocellular junctions) and/or indirect (chemical, paracrine/juxtacrine signalling, microvesicles and exosomes, sex hormone and microRNAs) contacts with various adjacent cells 48,49. TCs-mediated function-specific intercellular signalling contributes to regulate activity of neighbouring cells, including involvement in neurotransmission by spreading the slow waves generated by the pacemaker ICC 44, modulating tissue development/remodelling/metabolism, immunoregulation/immunosurveillance and maintaining oviduct homeostasis. In addition, oviduct TCs express estrogen/progesterone receptors, and thus might act as ‘hormonal sensors’, and their function was also, in part, under hormonal control 27.

Then, ultrastructure damage, loss of TCs (Fig.6A–E) and elevated level of estrogen (Fig.3D) will inevitably alter multiple intercellular signalling and cause associated problems. Generally, the female reproductive tract possesses a unique immune microenvironment, which tolerates the semi-allogeneic sperm and foetus, and protects against harmful pathogens 25,26. Meanwhile, as we know, MCs were known to be multifunctional players in local immune system. Uncontrolled augmentations in quantity, and/or activation of MCs can not only change SMC motility, microcirculation and contribute to sensation of pelvic pain or hyperalgesia in EMs 50, but also lead to pregnancy complications, such as miscarriage, tubal infertility and tubal ectopic pregnancy 51,52. Therefore, impaired MCs-mediated innate immunity (activation or repression) causes associated oviduct dysfunction or pelvic pain/dysmenorrhoea in EMs, involved in oviduct dysmotility, further influences intratubal fertilization process and contributed to EMs-associated fertility problems. On the other hand, local abnormal immunosurveillance will also influence the capability to destroy and prevent cancer cells from multiplying and forming tumours.

Secondly, the existence of SC niches, which were supported or guarded by TCs and their Tps, with intercellular connections between them (Fig.6E), indicated that oviduct TCs might play a role in recruitment of ancillary cells from the circulation into SC niches, nursing the development of adjacent SC and modulating its proliferative potential, thus participating in oviduct tissue repair/regeneration process. Similarly, TCs-supported function-specific SC niches were also reported in human skin, cardiac and eye tissue 8,14,15,28,49,53–57. On the other hand, according to the ‘bulge activation hypothesis’, SC will proliferate and differentiate only after receiving different environmental signals from specialized adjacent stromal cells 58. Thus, based on these tandem TCs-SCs and specific cell contacts (Fig.6E) in present study (Fig.6D) and previous literatures 48,49, we suggested that such a heterocellular mixture was more effective in potentially modulating the activity of SC-mediated tissue repair/regeneration processes. However, damage and loss of TCs will change the activity of TCs-SCs and decrease tissue reparation or renewal capacity, subsequently inducing development of tissue fibrosis. Continuous TCs loss and progressive interstitial fibrosis within EMs-affected oviduct (Fig.6B, D and E) will then cause oviduct dysfunction and fertility problems.

Thirdly, TCs usually embedded into bundles of collagen and elastic fibres, connected or combined different structural components of interstitium into an integrate system with their extremely long and thin Tps in present study and previous literatures 29,31,49,54,59. Thus, correctly constructed or organized a unique 3-D extracellular matrix of the connective tissue within organs. Such 3-D structure morphologically seems to be highly dependent on TCs and their Tps, function to not only guide directional distribution and migration of other cells (including SC), provide mechanical supporting structure for tissue growth/morphogenesis/angiogenesis and maintenance 29,44, but also act as a key regulator of integrating the function of neurotransmission and possibly contributing to spread the slow waves generated by the ICC, regulating the activity of neighbouring cells (SC, immunocytes, *etc*.), with intercellular different signalling mechanisms 26,48,49. Therefore, damage or loss of TCs might disturb their spatial relationships with adjacent multicellular entities, which involves extensive structural and molecular changes. TCs damage and 3-D interstitial architectural derangement will then impair their interconnecting role and cause loss of control of various adjacent cells and capillaries, which was the basis for intercellular signalling, self-repair, tissue homeostasis and angiogenesis. Especially, the activation of fibrocytes and their transition to myofibroblasts can contribute to fibrotic remodelling, progressively leading to interstitial fibrosis (Figs1 and 6B, D, E), as it has been recently proposed in fibrotic lesions of skin 29,30, Crohn's disease 31 and experimental myocardial infarction 28. This finally causes oviduct dysfunction and consequent reproductive problems. Nevertheless, TCs with nearly normal appearance can also be found in disease-affected tissue (Fig.6D); this might explain that, in some cases, EMs is associated with sub- or reduced fertility rather than absolute infertility.

Taken together, although the exact function(s) of TCs is (are) still not well defined, our results demonstrated a broad involvement of TCs, accompanied by the fibrotic remodelling in EMs-affected oviduct wall and might contribute to derangement of tissue architecture: (*i*) dysregulation of intercellular signalling, including immunoregulation/immunosurveillance, pacemaker activity, pelvic pain of EMs, (*ii*) impaired SC-mediated oviduct repair and/or regeneration capacity, and formation of interstitial fibrosis, (*iii*) impaired 3-D extracellular architecture reorganization, which were structural basis for intercellular signalling, tissue repair/remodelling and homeostasis.

Nevertheless, in EMs, what exactly happened on the pathway, by which TCs mediate cell interactions with other structural components of oviduct, remains undefined. And in future steps, animal models with defects in oviduct TCs networks still need to be developed to clarify the real functional consequences of TCs damage on reproductive activities. Also, it would be more valuable to perform high-throughput technologies such as comparative proteomics and bioinformatics analysis between disease-affected and -unaffected tissue, to provide new insights into potential roles of TCs in oviduct pathophysiology. And, additionally, whether TCs damage simultaneously occurred at uterine level and affects fertility capacity in EMs may be another critical issue. Finally, like emerging treatments aimed at promoting regeneration and reparation of injury-induced acute myocardial infarction by using TCs transportation 28, we suggest that TCs provide a new choice; rebuilding TCs network might be of great value for structural and functional reparation of fibrotic disease of oviduct.

## Supporting Information

**Figure S1** Representative single-labeled immunofluorescence image of iNOS in EMs-affected and -unaffected oviduct tissue (original magnification, 100×).

**Figure S2** Representative single-labeled immunofluorescence image of COX-2 in EMs-affected and -unaffected oviduct tissue (original magnification, 100×).

**Figure S3** Representative single-labeled immunofluorescence image of LPO in EMs-affected and -unaffected oviduct tissue (original magnification, 100×).

**Figure S4** Representative single-labeled immunofluorescence image of Estradiol in EMs-affected and -unaffected oviduct tissue (original magnification, 100×).

## References

[b1] Burney RO, Giudice LC (2012). Pathogenesis and pathophysiology of endometriosis. Fertil Steril.

[b2] Ren Q-Z, Qian Z-H, Jia S-H (2011). Vascular endothelial growth factor expression up-regulated by endometrial ischemia in secretory phase plays an important role in endometriosis. Fertil Steril.

[b3] Macer ML, Taylor HS (2012). Endometriosis and infertility: a review of the pathogenesis and treatment of endometriosis-associated infertility. Obstet Gynecol Clin North Am.

[b4] Mier-Cabrera J, Jiménez-Zamudio L, García-Latorre E (2011). Quantitative and qualitative peritoneal immune profiles, T-cell apoptosis and oxidative stress-associated characteristics in women with minimal and mild endometriosis. BJOG Int J Obstet Gy.

[b5] Czernobilsky B, Silverstein A (1978). Salpingitis in ovarian endometriosis. Fertil Steril.

[b6] Kissler S, Zangos S, Wiegratz I (2007). Utero-tubal sperm transport and its impairment in endometriosis and adenomyosis. Ann N Y Acad Sci.

[b7] Popescu LM, Manole CG, Gherghiceanu M (2010). Telocytes in human epicardium. J Cell Mol Med.

[b8] Gherghiceanu M, Popescu LM (2010). Cardiomyocyte precursors and telocytes in epicardial stem cell niche: electron microscope images. J Cell Mol Med.

[b9] Rusu MC, Pop F, Hostiuc S (2012). Telocytes form networks in normal cardiac tissues. Histol Histopathol.

[b10] Pieri L, Vannucchi MG, Faussone-Pellegrini MS (2008). Histochemical and ultrastructural characteristics of an interstitial cell type different from ICC and resident in the muscle coat of human gut. J Cell Mol Med.

[b11] Cretoiu D, Cretoiu SM, Simionescu AA (2012). Telocytes, a distinct type of cell among the stromal cells present in the lamina propria of jejunum. Histol Histopathol.

[b12] Zheng Y, Li H, Manole CG (2011). Telocytes in trachea and lungs. J Cell Mol Med.

[b13] Zheng Y, Bai C, Wang X (2012). Potential significance of telocytes in the pathogenesis of lung diseases. Expert Rev Respir Med.

[b14] Popescu LM, Gherghiceanu M, Suciu LC (2011). Telocytes and putative stem cells in the lungs: electron microscopy, electron tomography and laser scanning microscopy. Cell Tissue Res.

[b15] Popescu L, Manole E, Şerboiu CS (2011). Identification of telocytes in skeletal muscle interstitium: implication for muscle regeneration. J Cell Mol Med.

[b16] Qi G, Lin M, Xu M (2012). Telocytes in the human kidney cortex. J Cell Mol Med.

[b17] Gevaert T, Vos R, Aa F (2012). Identification of telocytes in the upper lamina propria of the human urinary tract. J Cell Mol Med.

[b18] Hinescu ME, Gherghiceanu M, Suciu L (2011). Telocytes in pleura: two-and three-dimensional imaging by transmission electron microscopy. Cell Tissue Res.

[b19] Nicolescu MI, Popescu LM (2012). Telocytes in the interstitium of human exocrine pancreas: ultrastructural evidence. Pancreas.

[b20] Nicolescu MI, Bucur A, Dinca O (2012). Telocytes in parotid glands. Anat Rec.

[b21] Gherghiceanu M, Popescu L (2005). Interstitial Cajal-like cells (ICLC) in human resting mammary gland stroma. Transmission electron microscope (TEM) identification. J Cell Mol Med.

[b22] Suciu L, Popescu LM, Gherghiceanu M (2010). Telocytes in human term placenta: morphology and phenotype. Cells Tissues Organs.

[b23] Hatta K, Huang ML, Weisel RD (2012). Culture of rat endometrial telocytes. J Cell Mol Med.

[b24] Cretoiu S, Simionescu A, Caravia L (2011). Complex effects of imatinib on spontaneous and oxytocin-induced contractions in human non-pregnant myometrium. Acta Physiol Hung.

[b25] Popescu L, Ciontea SM, Cretoiu D (2005). Novel type of interstitial cell (Cajal-like) in human fallopian tube. J Cell Mol Med.

[b26] Popescu LM, Ciontea SM, Cretoiu D (2007). Interstitial Cajal-like cells in human uterus and fallopian tube. Ann N Y Acad Sci.

[b27] Cretoiu SM, Cretoiu D, Suciu L (2009). Interstitial Cajal-like cells of human Fallopian tube express estrogen and progesterone receptors. J Mol Hist.

[b28] Zhao B, Chen S, Liu J (2013). Cardiac telocytes were decreased during myocardial infarction and their therapeutic effects for ischaemic heart in rat. J Cell Mol Med.

[b29] Manetti M, Guiducci S, Ruffo M (2013). Evidence for progressive reduction and loss of telocytes in the dermal cellular network of systemic sclerosis. J Cell Mol Med.

[b30] Manetti M, Rosa I, Messerini L (2014). A loss of telocytes accompanies fibrosis of multiple organs in systemic sclerosis. J Cell Mol Med.

[b31] Milia AF, Ruffo M, Manetti M (2013). Telocytes in Crohn's disease. J Cell Mol Med.

[b32] Yang X-J, Xu J-Y, Shen Z-J (2013). Immunohistochemical alterations of cajal-like type of tubal interstitial cells in women with endometriosis and tubal ectopic pregnancy. Arch Gynecol Obstet.

[b33] Yang XJ, Wei W, Zhao J (2012). Inhibitory effects of methotrexate on spontaneous motility and Cajal-like type of tubal interstitial cells in rabbit oviduct. Fertil Steril.

[b34] Berkley KJ, Dmitrieva N, Curtis KS (2004). Innervation of ectopic endometrium in a rat model of endometriosis. Proc Natl Acad Sci USA.

[b35] Vernon MW, Wilson E (1985). Studies on the surgical induction of endometriosis in the rat. Fertil Steril.

[b36] Appleyard CB, Cruz ML, Rivera E (2007). Experimental endometriosis in the rat is correlated with colonic motor function alterations but not with bacterial load. Reprod Sci.

[b37] Quereda F, Barroso J, Acien P (1996). Individual and combined effects of triptoreline and gestrinone on experimental endometriosis in rats. Eur J Obstet Gynecol Reprod Biol.

[b38] Fijneman RJ, Vos M, Berkhof J (2004). Genetic analysis of macrophage characteristics as a tool to identify tumor susceptibility genes mapping of three macrophage-associated risk inflammatory factors, marif1, marif2, and marif3. Cancer Res.

[b39] Mandache E, Popescu L, Gherghiceanu M (2007). Myocardial interstitial Cajal-like cells (ICLC) and their nanostructural relationships with intercalated discs: shed vesicles as intermediates. J Cell Mol Med.

[b40] Dixon RE, Hwang SJ, Hennig GW (2009). Chlamydia infection causes loss of pacemaker cells and inhibits oocyte transport in the mouse oviduct. Biol Reprod.

[b41] Duquette R, Shmygol A, Vaillant C (2005). Vimentin-positive, c-kit-negative interstitial cells in human and rat uterus: a role in pacemaking?. Biol Reprod.

[b42] Ciontea SM, Radu E, Regalia T (2005). C-kit immunopositive interstitial cells (Cajal-type) in human myometrium. J Cell Mol Med.

[b43] Vanderwinden J-M, Rumessen JJ, De Laet M (1999). CD34+ cells in human intestine are fibroblasts adjacent to, but distinct from, interstitial cells of Cajal. Lab Invest.

[b44] Pellegrini M-SF, Popescu LM (2011). Telocytes. Biomol Concept.

[b45] Zheng Y, Zhang M, Qian M (2013). Genetic comparison of mouse lung telocytes with mesenchymal stem cells and fibroblasts. J Cell Mol Med.

[b46] Zheng Y, Cretoiu D, Yan G (2014). Comparative proteomic analysis of human lung telocytes with fibroblasts. J Cell Mol Med.

[b47] Cretoiu SM, Cretoiu D, Marin A (2013). Telocytes: ultrastructural, immunohistochemical and electrophysiological characteristics in human myometrium. Reproduction.

[b48] Smythies J, Edelstein L (2013). Telocytes, exosomes, gap junctions and the cytoskeleton: the makings of a primitive nervous system?. Front Cell Neurosci.

[b49] Ceafalan L, Gherghiceanu M, Popescu L (2012). Telocytes in human skin–are they involved in skin regeneration?. J Cell Mol Med.

[b50] Anaf V, Chapron C, El Nakadi I (2006). Pain, mast cells, and nerves in peritoneal, ovarian, and deep infiltrating endometriosis. Fertil Steril.

[b51] Woidacki K, Zenclussen AC, Siebenhaar F (2014). Mast cell-mediated and associated disorders in pregnancy: a risky game with an uncertain outcome?. Front Immunol.

[b52] Sandvei R, Anker C, Wollen AL (1986). Mast cells in the tubal wall in women using an intrauterine contraceptive device. BJOG-Int J Obstet Gy.

[b53] Zheng Y, Bai C, Wang X (2012). Telocyte morphologies and potential roles in diseases. J Cell Physiol.

[b54] Bani D, Formigli L, Gherghiceanu M (2010). Telocytes as supporting cells for myocardial tissue organization in developing and adult heart. J Cell Mol Med.

[b55] Gherghiceanu M, Popescu LM (2012). Cardiac telocytes—their junctions and functional implications. Cell Tissue Res.

[b56] Popescu L, Gherghiceanu M, Manole C (2009). Cardiac renewing: interstitial Cajal-like cells nurse cardiomyocyte progenitors in epicardial stem cell niches. J Cell Mol Med.

[b57] Luesma MJ, Gherghiceanu M, Popescu LM (2013). Telocytes and stem cells in limbus and uvea of mouse eye. J Cell Mol Med.

[b58] Sun T-T, Costsarelis G, Lavker RM (1991). Hair follicular stem cells: the bulge-activation hypothesis. J Invest Dermatol.

[b59] Faussone-Pellegrini MS, Bani D (2010). Relationships between telocytes and cardiomyocytes during pre-and post-natal life. J Cell Mol Med.

