# Supplementary figures and images for: Telocytes damage in endometriosis-affected rat oviduct and potential impact on fertility
Source: J Cell Mol Med. 2014 Nov 11;19(2):452–62. doi: 10.1111/jcmm.12427 (PMC4407595; doi:10.1111/jcmm.12427)

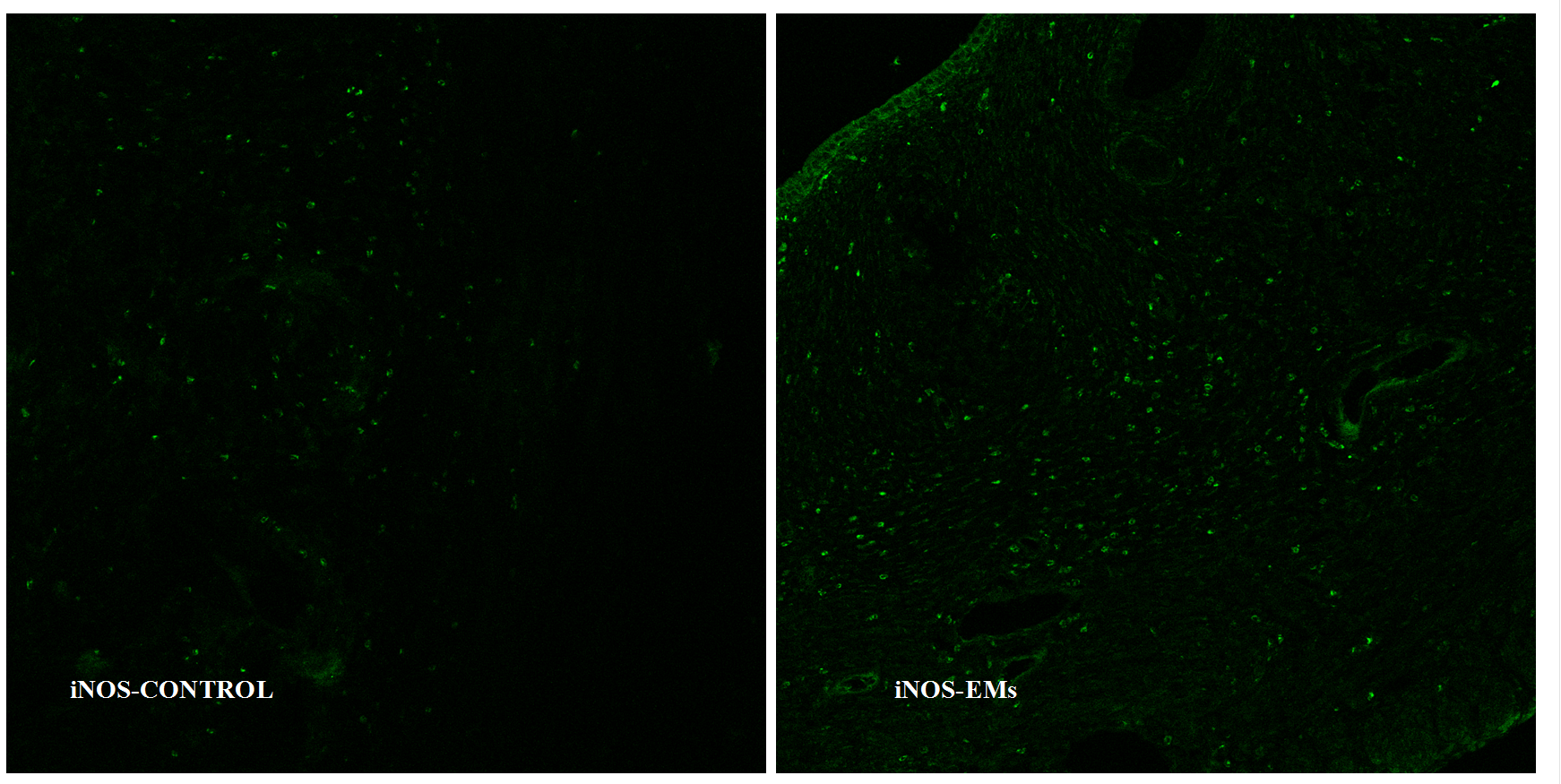

Supplement: Supplementary file 1 [file jcmm0019-0452-sd1.tif]

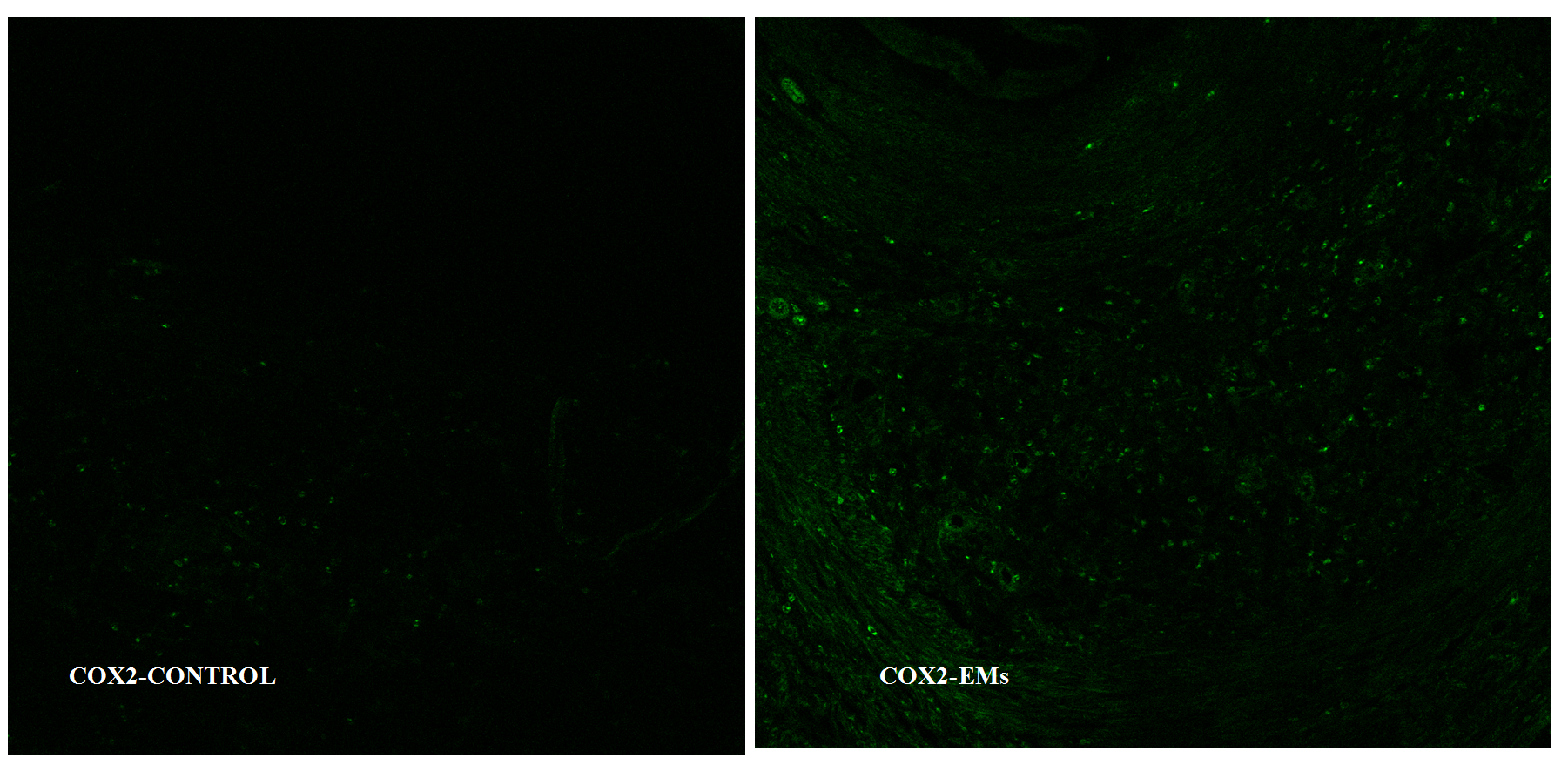

Supplement: Supplementary file 2 [file jcmm0019-0452-sd2.tif]

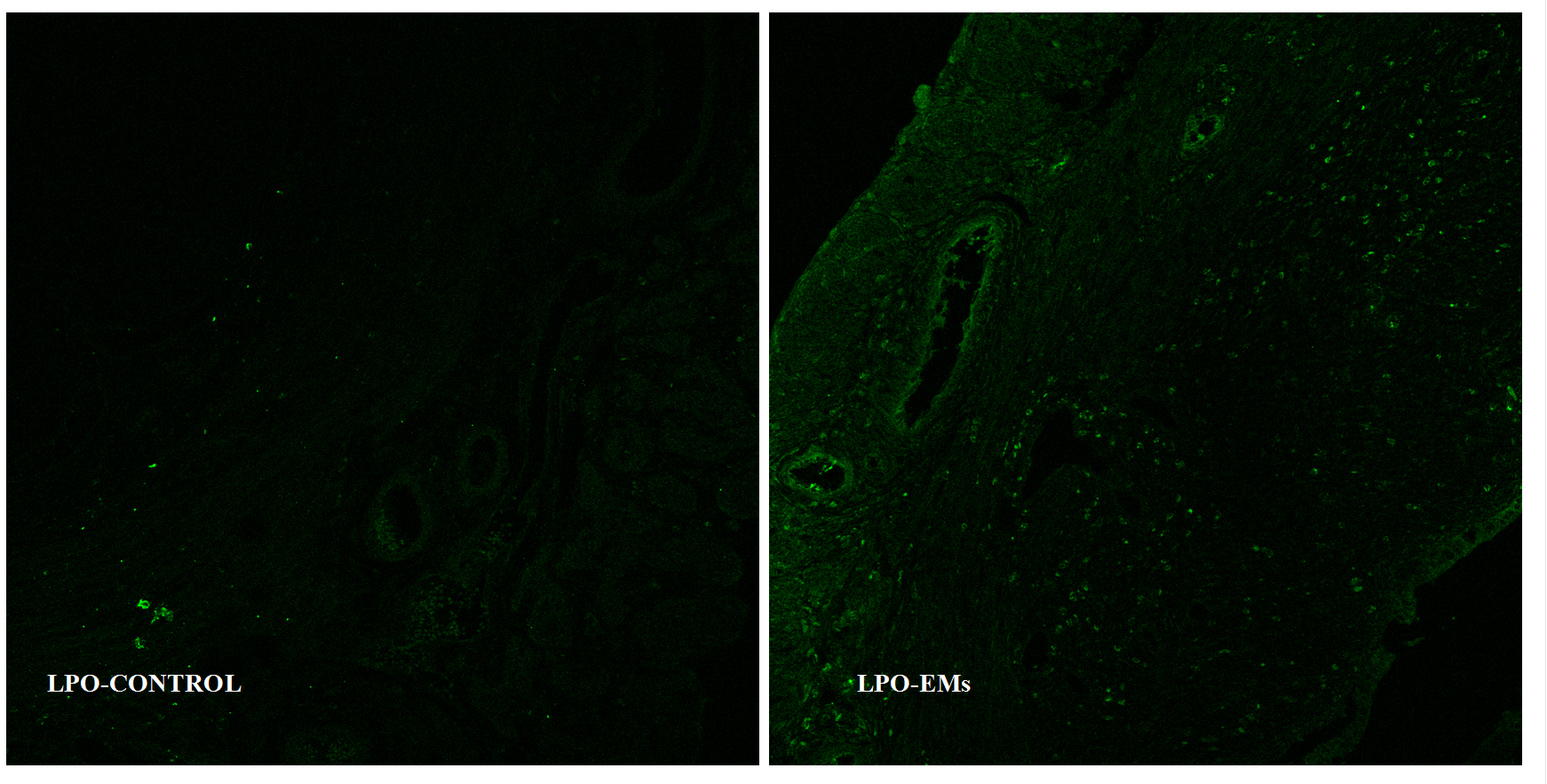

Supplement: Supplementary file 3 [file jcmm0019-0452-sd3.tif]

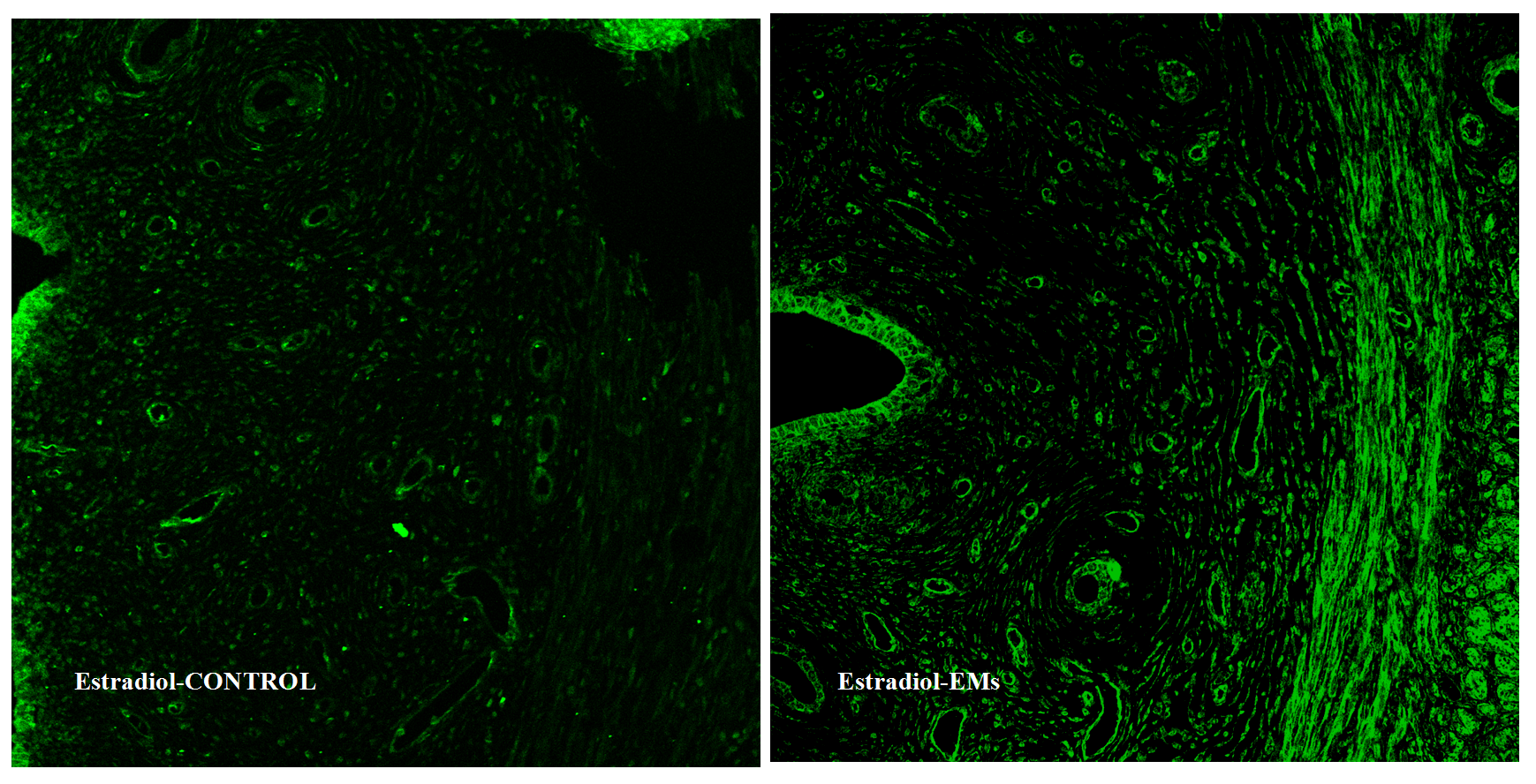

Supplement: Supplementary file 4 [file jcmm0019-0452-sd4.tif]
